# Testis cell pyroptosis mediated by *CASP1* and *CASP4*: possible sertoli cell-only syndrome pathogenesis

**DOI:** 10.1186/s12958-023-01101-w

**Published:** 2023-06-09

**Authors:** Wantao Liu, Xinan Li, Qiang Ma, Yongtong Zhu, Wenzhong Zhao, Yisheng Yang, Weiqiang Xiao, Daxiong Huang, Fengbo Cai, David Yiu Leung Chan, Shanchao Zhao, Qingjun Chu

**Affiliations:** 1 The First School of Clinical Medicine, Nanfang Hospital, Southern Medical University, Guangzhou, China; 2 School of Basic Medical Sciences, Guangzhou Medical University, Guangzhou, 511436 People’s Republic of China; 3 NHC Key Laboratory of Male Reproduction and Genetics, Family Planning Research Institute of Guangdong Province, Guangzhou, China; 4 Assisted Reproductive Technology Unit, Department of Obstetrics and Gynaecology, Faculty of Medicine, The Chinese University of Hong Kong, Prince of Wales Hospital, Hong Kong, China

**Keywords:** Sertoli cell-only syndrome (SCOS), CASP1, CASP4, Pyroptosis

## Abstract

**Background:**

Sertoli cell-only syndrome (SCOS) is the most serious pathological type of non-obstructive azoospermia. Recently, several genes related to SCOS have been identified, including *FANCM*, *TEX14*, *NR5A1*, *NANOS2*, *PLK4*, *WNK3*, and *FANCA*, but they cannot fully explain the pathogenesis of SCOS. This study attempted to explain spermatogenesis dysfunction in SCOS through testicular tissue RNA sequencing and to provide new targets for SCOS diagnosis and therapy.

**Methods:**

We analyzed differentially expressed genes (DEGs) based on RNA sequencing of nine patients with SCOS and three patients with obstructive azoospermia and normal spermatogenesis. We further explored the identified genes using ELISA and immunohistochemistry.

**Results:**

In total, 9406 DEGs were expressed (Log2|FC|≥ 1; adjusted *P* value < 0.05) in SCOS samples, and 21 hub genes were identified. Three upregulated core genes were found, including *CASP4*, *CASP1*, and *PLA2G4A*. Thus, we hypothesized that testis cell pyroptosis mediated by *CASP1* and *CASP4* might be involved in SCOS occurrence and development. ELISA verified that CASP1 and CASP4 activities in the testes of patients with SCOS were significantly higher than those in patients with normal spermatogenesis. Immunohistochemical results showed that CASP1 and CASP4 in the normal spermatogenesis group were mainly expressed in the nuclei of spermatogenic, Sertoli, and interstitial cells. CASP1 and CASP4 in the SCOS group were mainly expressed in the nuclei of Sertoli and interstitial cells because of the loss of spermatogonia and spermatocytes. CASP1 and CASP4 expression levels in the testes of patients with SCOS were significantly higher than those in patients with normal spermatogenisis. Furthermore, the pyroptosis-related proteins GSDMD and GSDME in the testes of patients with SCOS were also significantly higher than those in control patients. ELISA also showed that inflammatory factors (IL-1 β, IL-18, LDH, and ROS) were significantly increased in the SCOS group.

**Conclusions:**

For the first time, we found that cell pyroptosis-related genes and key markers were significantly increased in the testes of patients with SCOS. We also observed many inflammatory and oxidative stress reactions in SCOS. Thus, we propose that testis cell pyroptosis mediated by *CASP1* and *CASP4* could participate in SCOS occurrence and development.

**Supplementary Information:**

The online version contains supplementary material available at 10.1186/s12958-023-01101-w.

## Background

Male infertility is a common reproductive disorder that contributes about 10–15% of global infertility [1–4], and is becoming an increasingly prevalent problem for couples of childbearing age worldwide. Many factors can lead to male infertility. Azoospermia is a severe form of male infertility which consists of obstructive azoospermia (OA) and non-obstructive azoospermia (NOA) [5]. Azoospermia accounts for about 20–30% of male infertility. Azoospermia is defined as a lack of sperm in the semen after excluding non-ejaculation or retrograde ejaculation. OA refers to the absence of sperm and spermatogenic cells in semen owing to deferential duct obstruction. NOA is defined as testicular spermatogenesis dysfunction and accounts for 60% of azoospermia cases [6]; it is one of the most serious forms of azoospermia. With the development of medical science, scholars have made remarkable achievements in diagnosing and treating NOA. However, most patients with NOA still cannot become biological fathers through assisted reproductive technology.

Testicular biopsy remains the most accurate diagnostic method for evaluating testicular function in clinical practice, and can identify NOA and OA in infertile men. Most patients with NOA exhibit primary testicular failure and inherent defects in spermatogenesis. A few NOA cases involve secondary testicular failure caused by endocrine disorders or other testicular factors, such as developmental defects. Many NOA cases are congenital, and a considerable number of patients show genetic causes, such as abnormal chromosome karyotype (17%), pathogenic Y chromosome microdeletions (2–10%), and abnormal mutations in other genes. Sertoli cell-only syndrome (SCOS) is the type of NOA with the most severely impaired spermatogenesis. SCOS is usually determined via pathological diagnosis, which depends on puncture/incision biopsy [7, 8]. Testicular biopsy samples normally display seminiferous tubules lined with Sertoli cells, but no germ cells.

There are various known etiologies of human SCOS, including chromosome disorders (mostly Klinefelter syndrome), Y chromosome microdeletions, cryptorchidism, radiation, cytotoxic drugs, mumps orchitis, and other possible causes of testicular diseases, including varicocele, testicular hydrocele, and testicular microlithiasis. However, there are still many patients with SCOS caused by clinically unknown factors. Consequently, this study used RNA sequencing (RNA-seq) to explore the possible pathogenic genes and signaling pathways in SCOS caused by ambiguous factors, which excludes the abovementioned definite factors (Klinefelter syndrome, genomic AZF deletions, cryptorchidism, mumps orchitis, radiation, and cytotoxic drugs).

Spermatogenesis is a complex and well-organized process. However, its causes have not been fully clarified. Spermatogenesis occurs in the seminiferous tubules and relies on their appropriate microenvironment or niche [9, 10]. As primary support cells, Sertoli cells are involved in all stages of spermatogenesis and are believed to be critical to the process [10]. Proper gene expression patterns are the basis of Sertoli cell functioning and male germ cell differentiation. Abnormal Sertoli cell transcriptomes are associated with spermatogenesis dysfunction, which may lead to azoospermia in humans [5].

Although spermatogenesis has been intensely researched in recent years, many genes involved in this process remain uncertain. Detailed knowledge of molecular regulation in the testes at the transcriptional level is essential for understanding the complex interaction between normal and pathological conditions. Previous studies have identified genes that may be involved in the occurrence of SCOS, including *FANCM*, *TEX14*, *NR5A1*, *WT1*, *NANOS2*, *PLK4*, *WNK3*, and *FANCA* [11]. However, they cannot explain SCOS or assist in its diagnosis and treatment.

In recent years, the application of high-throughput and next-generation sequencing technology has led to a breakthrough in tumor detection. As a result, specific genes have been detected in many kinds of tumors, and targeted drugs have been developed for clinical use. However, the application of high-throughput sequencing in male infertility is still being explored. RNA-seq is a cost-effective and high-throughput method of analyzing the transcriptome in specific tissues or cells [12, 13]. According to testicular biopsy results, we used the testicular tissues of patients with SCOS as the experimental group and those of patients with OA and normal spermatogenesis as the control group, aiming to explain spermatogenesis dysfunction in SCOS through RNA-seq and to provide new targets for its diagnosis and therapy.

## Materials and methods

### Screening testicular tissue samples from patients with SCOS

We collected testicular tissues from patients with SCOS and patients with OA who had normal spermatogenic function in the male clinic of the reproductive center from 2016 to 2019. Patients with OA have normal spermatogenic function, as they only suffer from reproductive tract obstruction. SCOS was confirmed via histological analysis. Patients with congenital reproductive diseases, such as Klinefelter syndrome, genomic AZF deletions, or other conditions, including cryptorchidism, mumps orchitis, radiation, and cytotoxic drug use, were excluded from this study. Detailed characteristics of the samples used in this study are shown in Table 1. Based on testicular biopsy, nine patients with SCOS were selected as the experimental group, and three patients with OA and normal spermatogenic function were selected as the control group. This study has been reviewed by the Ethics Committee of Southern Medical University, China, which approved the performed experiments.

**Table 1:** Description of the patients in the study

Parameter	OA	SCOS	*P* value
Age (years)	28.67 ± 4.73	31.33 ± 5.24	0.4588
FSH (mIU/mL)	4.67 ± 0.83	29.00 ± 6.52	< 0.0001
LH (mIU/mL)	3.59 ± 0.46	16.16 ± 3.37	< 0.0001
T (mIU/mL)	4.27 ± 0.15	6.21 ± 5.92	0.3387
FT (mIU/mL)	15.97 ± 2.78	15.97 ± 4.40	1
INH-B (pg/mL)	119.74 ± 12.50	10.28 ± 0.83	0.0043
Volume (mL)	16.67 ± 2.89	8.44 ± 2.35	0.0193

### RNA extraction from testicular tissues

Testicular tissues were lysed in TRIzol reagent (Invitrogen, Carlsbad, CA, USA). Total RNA was extracted using a RNeasy kit (Qiagen, Hilden, Germany) and stored at − 80 ºC.

### Library construction for RNA sequencing

RNA quality was examined using a 2100 Bioanalyzer (Agilent), and quantitation was performed using a NanoDrop2000. RNA samples with an RNA integrity number ≥ 8 were selected for preparing the libraries. RNA-seq libraries were constructed using the TruSeq® RNA Library Prep Kit v2 (Illumina, San Diego, CA, USA). Paired-end sequencing (2 × 150 bp) of the libraries was performed using a HiSeq 2500 system (Illumina).

### Data analysis

We analyzed differentially expressed genes (DEGs) using edgeR (Mark D. Robinson, 2010) according to gene expression. DEGs were identified based on the criteria: false discovery rate ≤ 0.05 and fold-change (FC) ≥ 2.

### Key genes identified in DEG interaction networks

The DEG interaction network can succinctly and intuitively reflect gene–gene interactions, and it can identify key genes through its topology. The network is composed of nodes and edges. Nodes can be categorized as source and target nodes. A node is a DEG, and an edge is the interaction between genes. We constructed a DEG interaction network using the STRING database and identified key genes in the interaction network using the MCODE algorithm of Cytoscape software.

### RT-PCR and quantitative real-time PCR

RT-PCR was performed to examine the expression of key genes. PCR products were separated via electrophoresis on 1.2% agarose gels, and the gels were exposed to chemiluminescence (Chemi-Doc XRS, Bio-Rad, Hercules, CA). Real-time PCR reactions were performed using a Thermal Cycler Dice TM Real-time System (Takara) and a SYBR Premix Ex Taq TM reagents kit (Takara). Real-time PCR was used to verify the expression profiles of key genes detected in the interaction networks. The expression level values were normalized to those of ACTB as a control. Relative FCs of mRNA expression were calculated using the 2^−ΔΔCt^ method, and the values were expressed as 2^−ΔΔCt^.

### Functional enrichment analysis

Gene Ontology (GO) is an international standardized classification system of gene function, which provides a set of dynamically updated controlled vocabulary to comprehensively describe the attributes of genes and gene products in organisms. GO includes three ontology types, which represent the gene molecular function (MF), cellular component (CC), and biological process (BP). The basic GO unit is the term, and each term corresponds to an attribute.

### Pathway enrichment analysis

The Kyoto Encyclopedia of Genes and Genomes (KEGG) is the main public pathway database. The most important biochemical metabolism and signal transduction pathways involving DEGs can be determined by significant pathway enrichment. For example, pathways with a Q value ≤ 0.05 were defined as significantly enriched in DEGs. Thus, the most important biochemical metabolism and signal transduction pathways involving DEGs can be determined through significant pathway enrichment.

### Western blot analysis

Western blotting was performed according to a previously described method. Briefly, tissue samples were homogenized in RIPA lysis buffer (Beyotime, Shanghai, China) containing protease inhibitor at 4 °C and centrifuged at 12,000 × g for 15 min. The supernatant was aliquoted, and protein concentration was determined using a BCA kit (Beyotime, Shanghai, China). The following primary antibodies were used: GSDMD, GSDME, CASP1, and CASP4 (rabbit, 1:500; Cell Signaling Technology, Houston, TX, USA). In addition, anti-GAPDH antibodies (rabbit, 1:1000; Service, Wuhan, China) were selected as an internal reference. After primary antibody incubation, the membranes were washed with TBST and incubated with goat anti-rabbit secondary antibodies (LICOR, NE, USA) for 2 h at room temperature. Band intensities were quantified using Quantity One (Bio-Rad, CA, USA) software.

#### ELISA

Tissue extracts were first diluted 1000–10,000 times in assay buffer and added to the wells along with the standard. For conditioned water extracts, methanol eluates were dried, reconstituted in assay buffer, and assayed.

### Immunohistochemistry analysis

Testicular tissues were incubated with primary antibodies against CASP1 and CASP4 (rabbit, 1:200; ABclonal, Wuhan, China), stained with secondary antibodies, and observed under an optical microscope.

### Statistical analysis

The results are presented as the means ± SEM. One-way ANOVA was performed, followed by post hoc analysis for multi-group comparisons. *P* < 0.05 was considered statistically significant.

## Results

### DEGs in patients with SCOS

Original data filtering and quality control is particularly important in the process of data analysis. Sequence quality is mainly characterized by the proportion of the Q20/Q30 value. The sequencing base mass was > 30 in our study. We used RNA-seq expectation–maximization tools to quantify the expression of genes and transcripts after quality control of the sequencing data was performed. Based on the quantitative results in fragments per kilobase of transcript per million fragments mapped, we calculated the correlation between all samples. According to the standard recommended by the Encode plan, the square of the Paerson correlation coefficient of the two samples in biological repetition should be ≥ 0.92 under ideal sampling and experimental conditions. Finally, we used the statistical gene expression results of each sample for DEG screening and analysis.

**Fig. 1:**
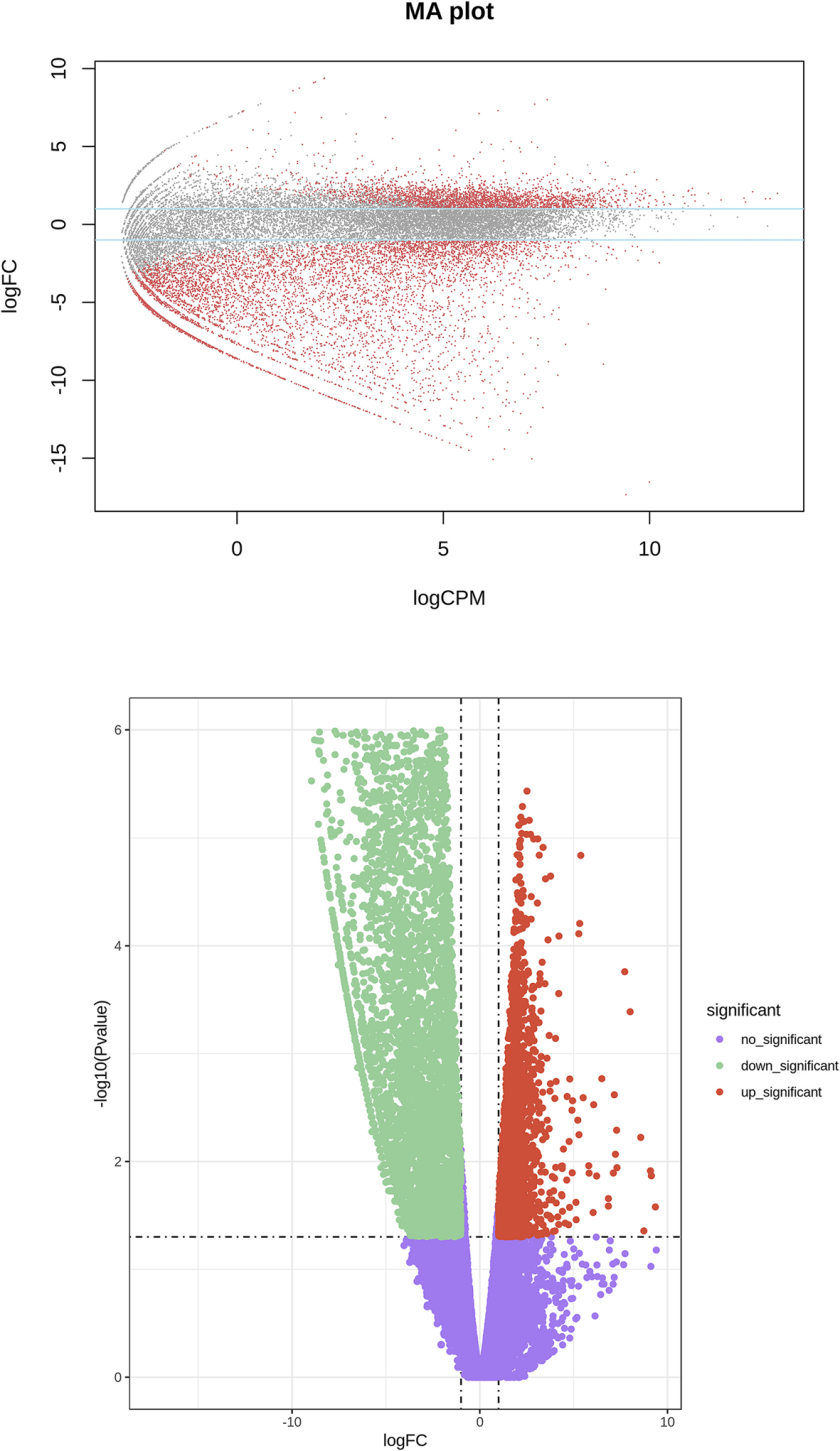
OA vs. SCOS. Volcano plot of differentially expressed genes. OA: obstructive azoospermia with normal spermatogenic function; SCOS: Sertoli cell-only syndrome

We analyzed DEGs in the SCOS and control groups using edgeR (Mark D. Robinson, 2010) and selected DEGs |log2Ratio|≥ 1 and *P* < 0.05. The results revealed 9406 DEGs, including 2541 upregulated genes and 6865 downregulated genes (Fig. 1).

### DEG interaction networks and key genes

**Fig. 2:**
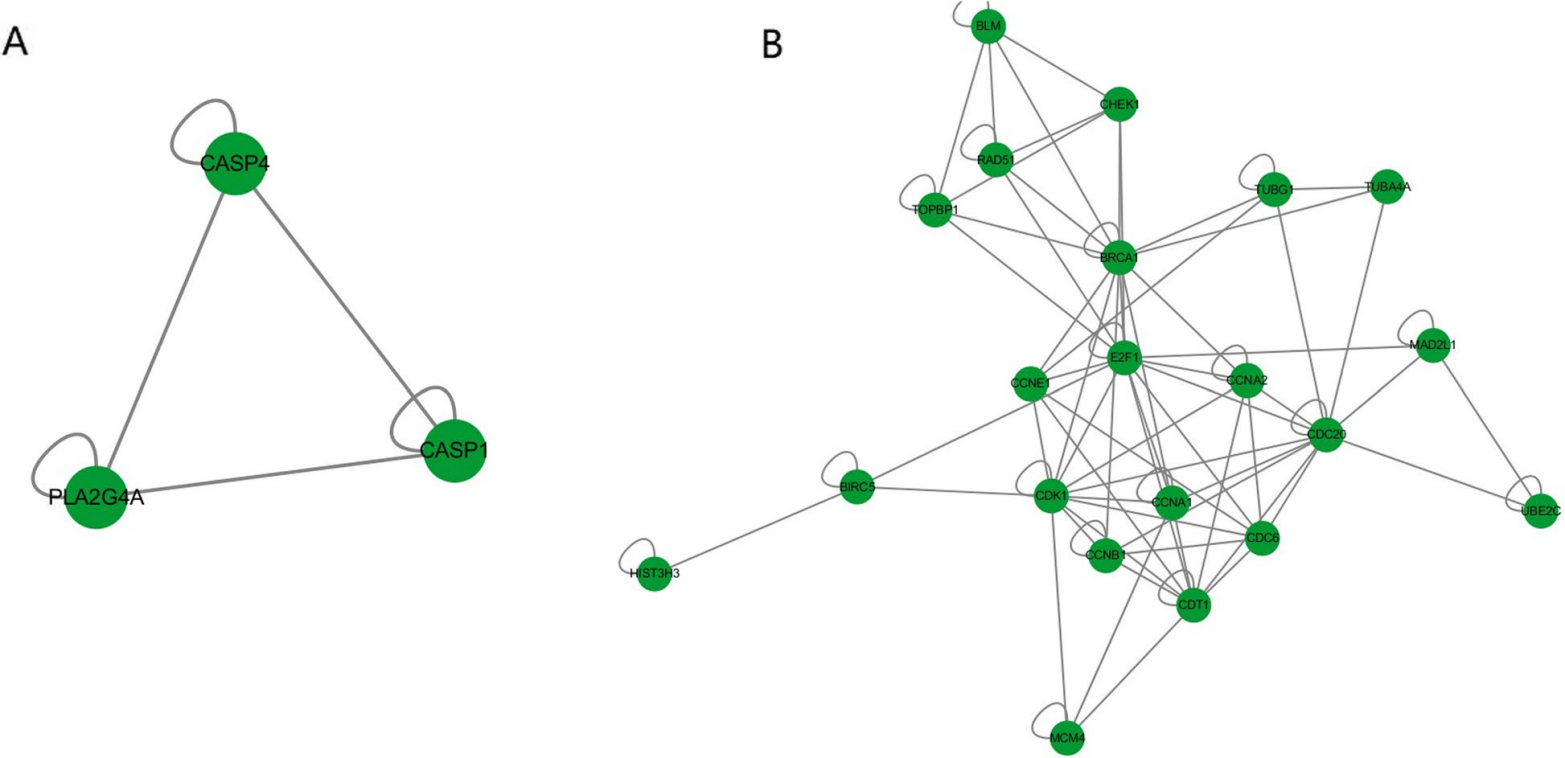
**A** OA vs. SCOS key genes (UP). OA: obstructive azoospermia with normal spermatogenic function; SCOS: Sertoli cell-only syndrome. **B** OA vs. SCOS key genes (DOWN) OA: obstructive azoospermia with normal spermatogenic function; SCOS: Sertoli cell-only syndrome

We constructed a PPI network based on the STRING online database and Cytoscape software to further explore the interplay between DEGs with prognostic value. Clustering analysis of the interaction network was carried out using Cytotype MCODE, and key genes were selected based on the degree of importance (Fig. 2A, B).

### Quantitative real-time PCR verification of key genes

**Fig. 3:**
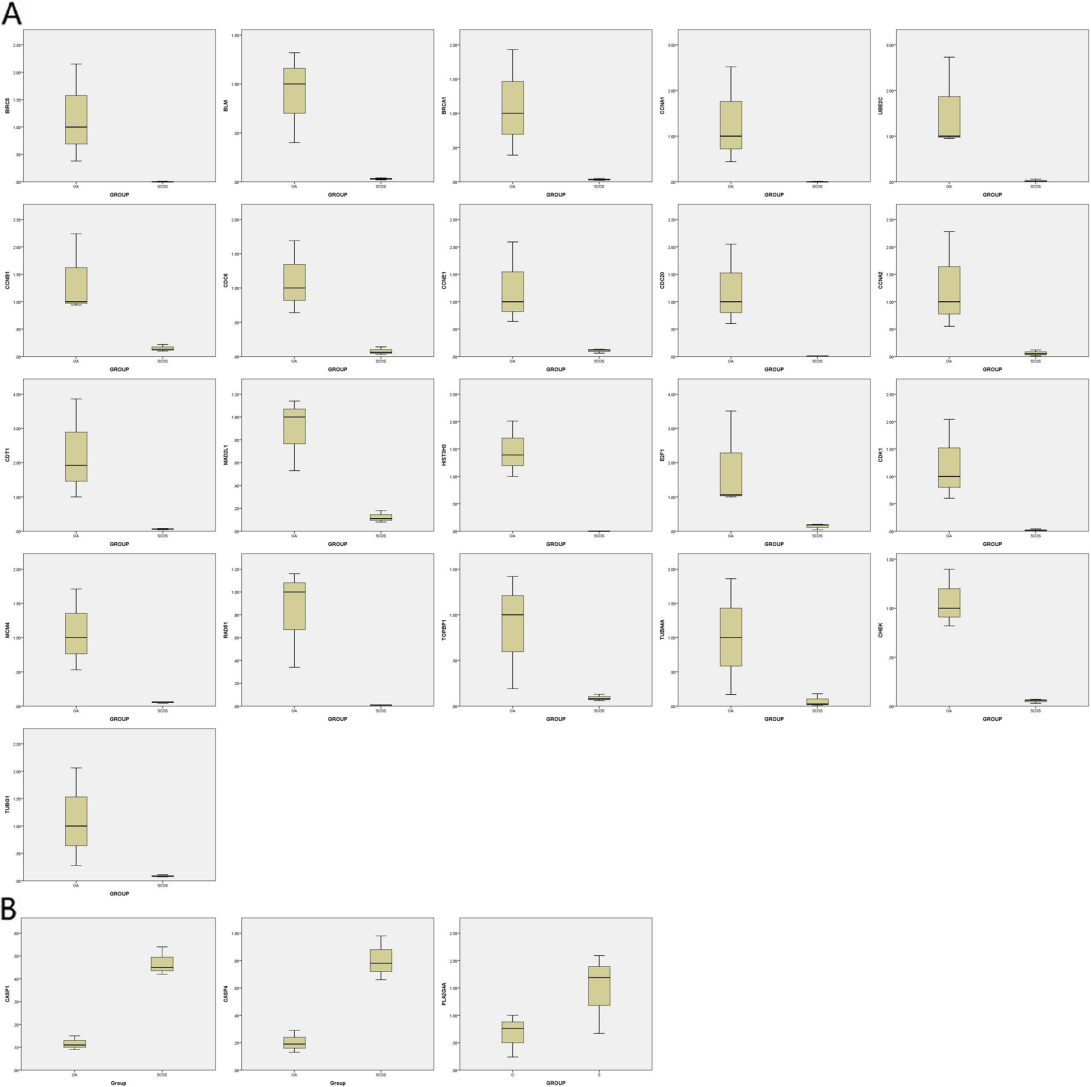
**A** Q-PCR verification of downregulated core differential genes (*P* < 0.05). **B** Q-PCR verification of upregulated core differential genes (*P* < 0.05)

To validate key genes, quantitative real-time PCR was performed to detect three upregulated and 21 downregulated genes. Quantitative real-time PCR revealed that *BRCA1*, *RAD51*, *BIRC5*, *CDC6*, *E2F1*, *MCM4*, *CDC20*, *TUBA4A*, *TUBG1*, *CCNA1*, *CCNB1*, *CCNA2*, *CHEK1*, *TOPBP1*, *MAD2L1*, *CDT1*, *HIST3H3*, *CDK1*, *BLM*, *CCNE1*, and *UBE2C* were significantly downregulated in patients with SCOS, compared with normal patients (Fig. 3A). In contrast, *CASP4*, *CASP1*, and *PLA2G4A* were significantly upregulated (Fig. 3B).

### Functional and pathway enrichment analysis

**Fig. 4:**
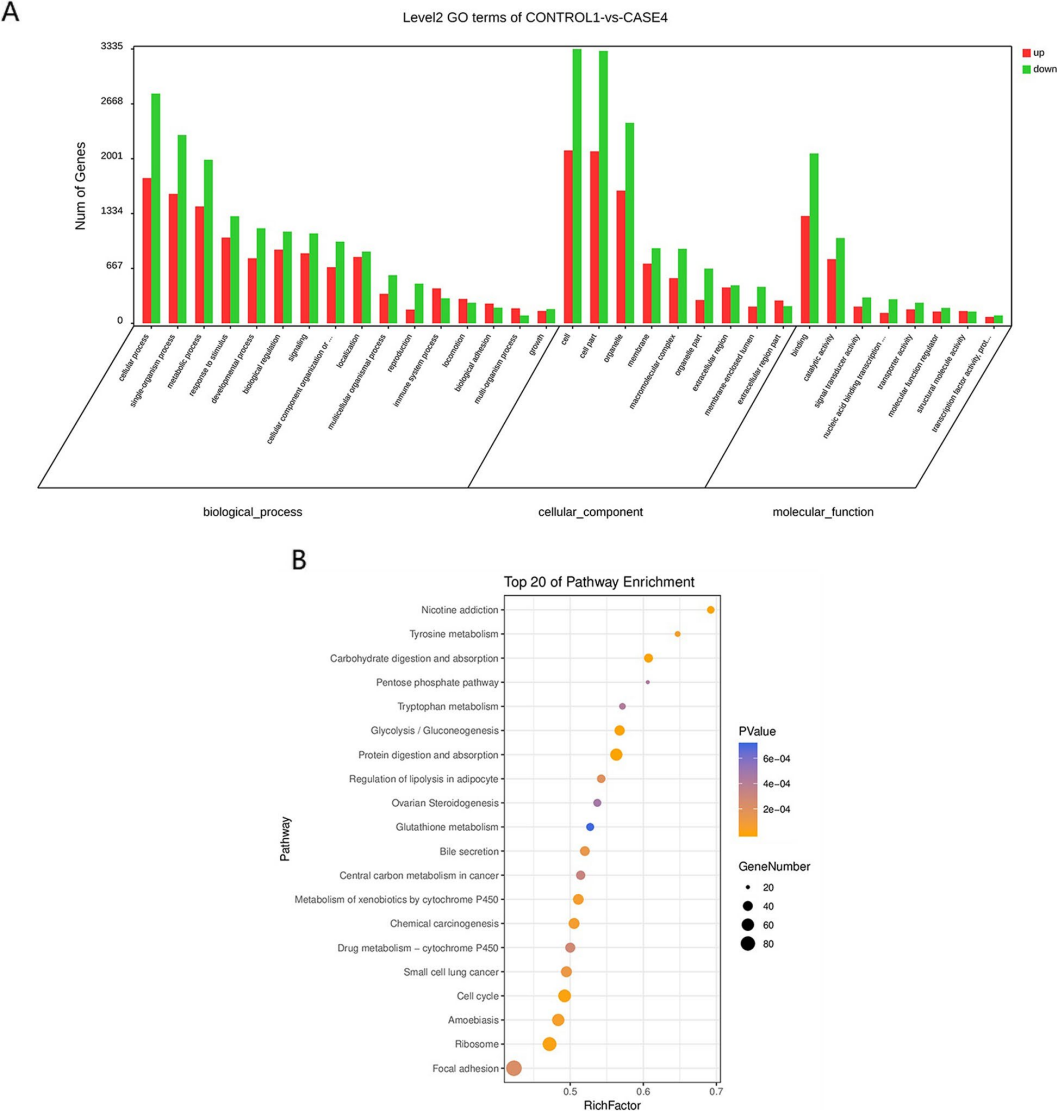
**A** Gene ontology (GO) enrichment of the differential expressed genes. GO analysis covered three types: biological process (BP), cellular component (CC), and molecular function (MF); red indicates upregulated genes; blue indicates downregulated genes. **B** Kyoto Encyclopedia of Genes and Genomes pathway enrichment in the differentially expressed genes. Dots represent term enrichment with color coding: red indicates high enrichment; blue indicates low enrichment. Dot sizes represent the percentage of each row

To estimate overrepresented genes in the functional class, dysregulated key genes were subjected to functional enrichment analysis. According to CC, most genes were categorized as nuclear part, intracellular organelle part, intracellular membrane-bounded organelle, and intracellular. Among BPs, most genes were enriched in the cell cycle, macromolecule metabolic process, cellular nitrogen compound metabolic process, and organelle organization. According to MF, genes were enriched in enzyme binding and nucleic acid binding (Fig. 4A). Five further pathways, including the cell cycle, progesterone-mediated oocyte maturation, oocytemeiosis, homologous recombination, and p53 signaling pathways, were enriched, based on KEGG analysis (Fig. 4B).

### GSDMD and GSDME pyroptosis results

**Fig. 5:**
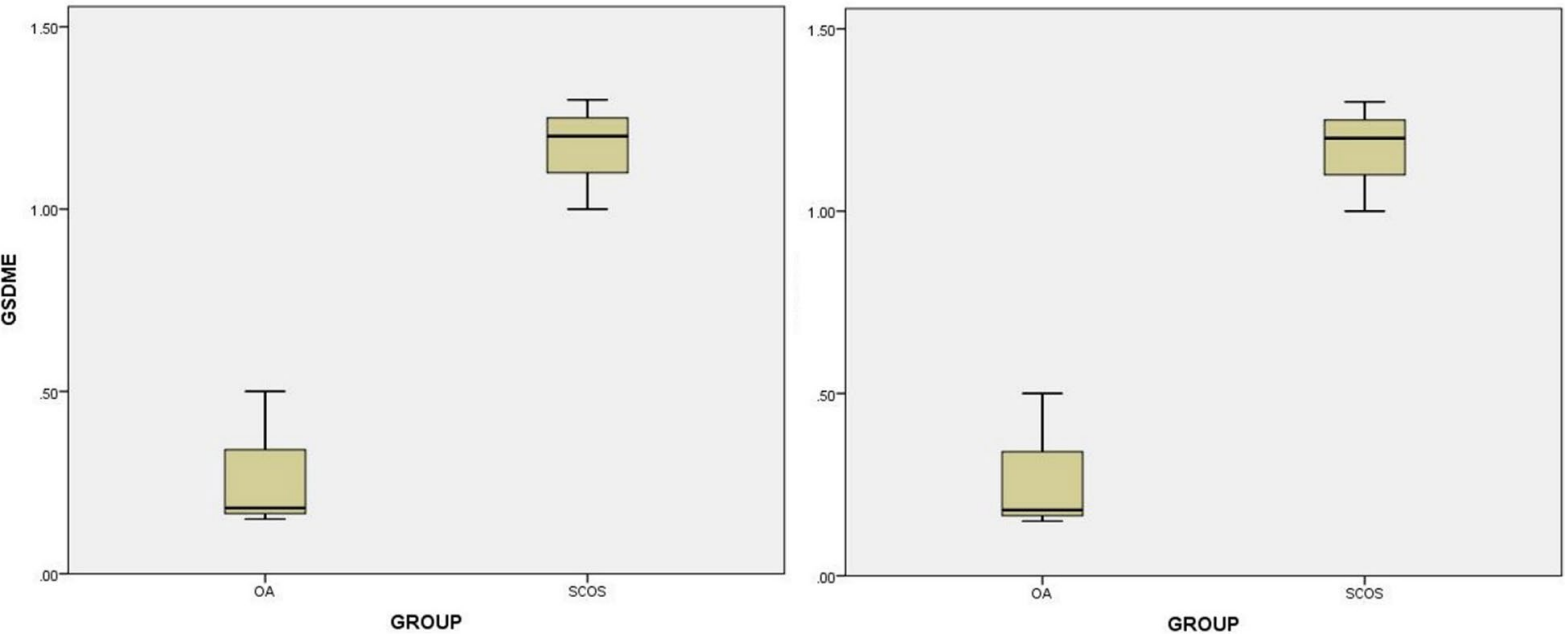
GSDMD and GSDME pyroptosis results (*P* < 0.05)

Activated CASP1 and CASP4 act on GSDMD/GSDME proteins and cleave them to pefrorate the membrane in the process of pyroptosis, which promotes cell dissolution and rupture. In this study, we used a q-PCR assay to analyze GSDMD and GSDME expression in fresh testicular tissues from patients with SCOS and normal spermatozoa. We found that GSDMD and GSDME expression in the testicular tissue from patients with SCOS was significantly higher than in tissue from patients with normal spermatogenesis (Fig. 5).

### CASP1 and CASP4 expression analysis

**Fig. 6:**
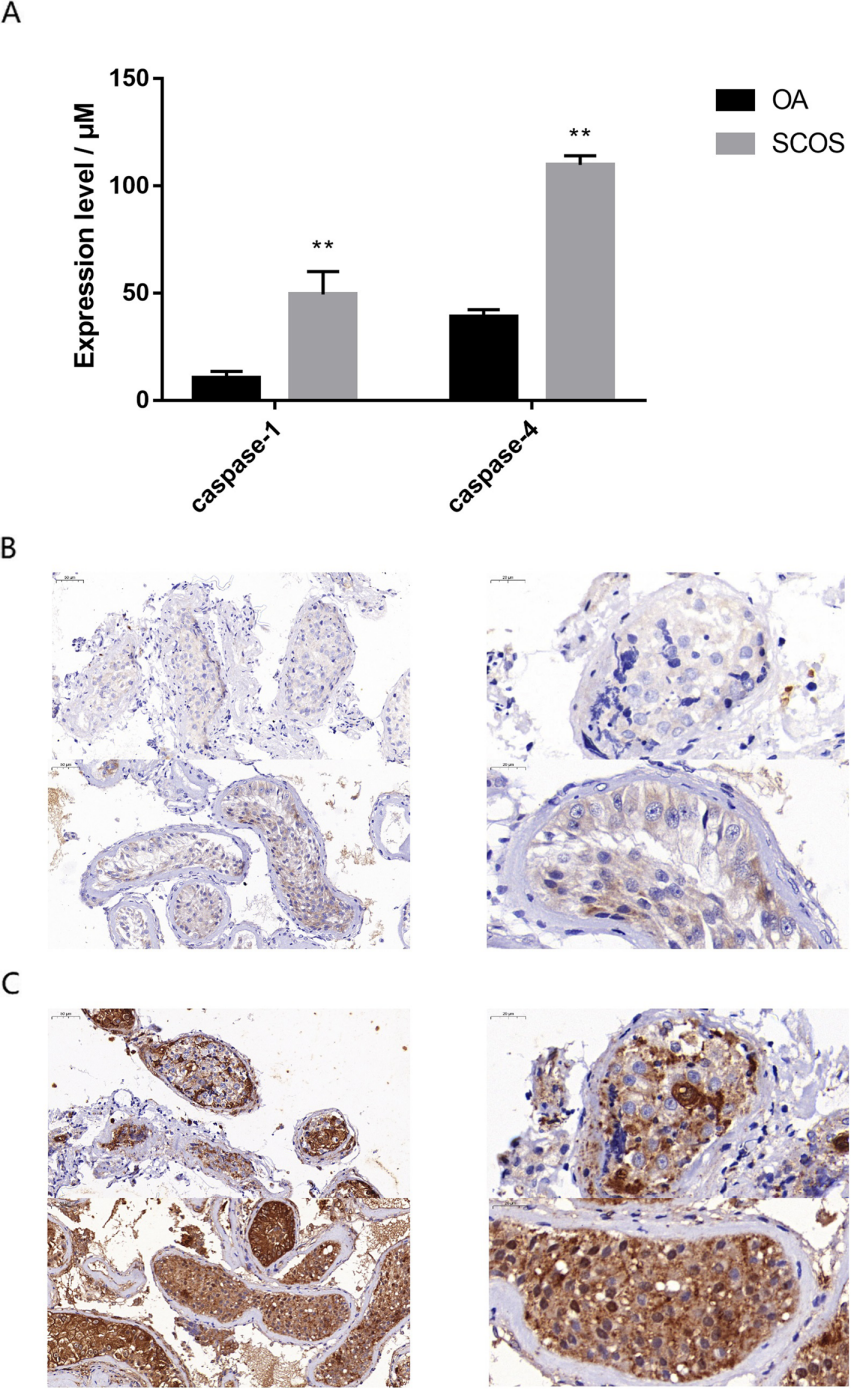
**A** Differences in CASP1 and CASP4 activity in the testicular tissues of patients with normal spermatogenesis and SCOS. OA: Normal spermatogenesis group; SCOS: Sertoli cell-only syndrome group; Asterisks (**) indicate significant differences at *P* < 0.01 between the treated and control groups. **B** CASP1 expression in the testes of patients with normal spermatogenesis (up) and Sertoli cell-only syndrome (down). (Left: 10 × ; Right: 20 ×). **C** CASP4 expression in the testes of patients with normal spermatogenesis (up) and Sertoli cell-only syndrome (down). (Left: 10 × ; Right: 20 ×)

In this study, we used visible light colorimetry to determine CASP1 and CASP4 activity. Through the experimental results, we found that their actions in the testes of patients with SCOS were significantly higher than those in patients with normal spermatogenesis (Fig. 6A).

In addition, the immunohistochemical results showed that CASP1 and CASP4 were mainly expressed in the nuclei of spermatogenic, Sertoli, and interstitial cells in the group with normal spermatogenesis. CASP1 and CASP4 were mainly expressed in the nuclei of spermatogenic and interstitial cells in the SCOS group, which may be caused by the loss of spermatogonia and spermatocytes. At the same time, CASP1 and CASP4 expression levels in the testes of patients with SCOS were significantly higher than in those of patients with normal spermatogenesis (Fig. 6B, C).

### Western blotting analysis of CASP1, CASP4, GSDMD, and GSDME

**Fig. 7:**
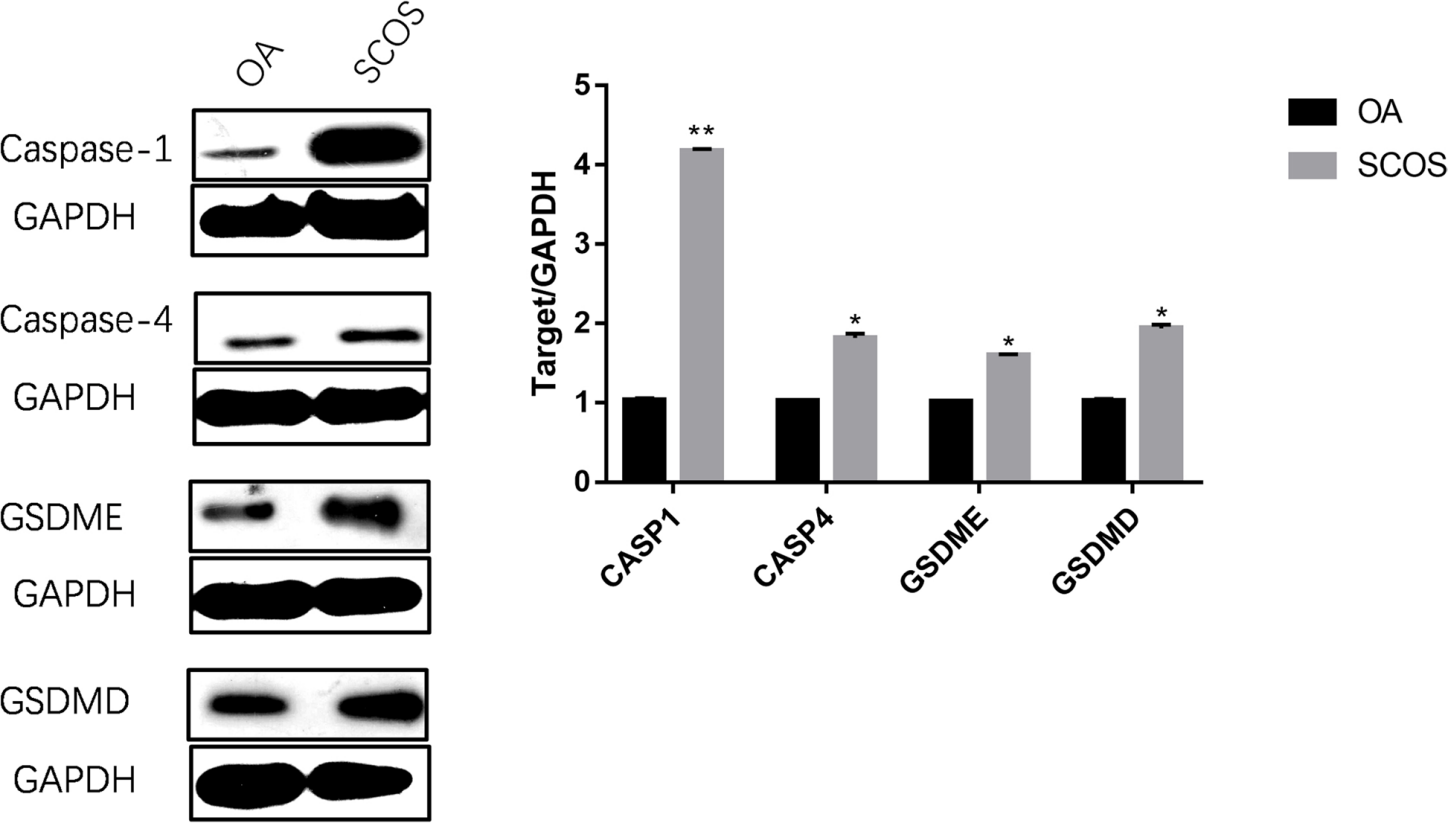
Differential analysis of protein expression of key genes in cell pyroptosis. OA: Normal spermatogenesis group; SCOS: Sertoli cell-only syndrome group; Asterisks (*) and (**) indicate significant differences at *P* < 0.05 and *P* < 0.01 between the MCeLR-treated and control groups, respectively (gels and blots in the original image made tailoring necessary; the relevant original documents can be seen in the Supplementary Materials)

To further demonstrate that CASP1, CASP4, GSDMD, and GSDME expression in the testes of patients with SCOS was significantly higher than in those with normal spermatogenesis, we used fresh testicular tissues for western blotting analysis. The results showed that CASP1, CASP4, GSDMD, and GSDME were significantly higher in the testicular tissue of patients with SCOS than in patients with normal spermatogenesis at the protein level (Fig. 7).

### Differential expression analysis of downstream cell pyroptosis products

**Fig. 8:**
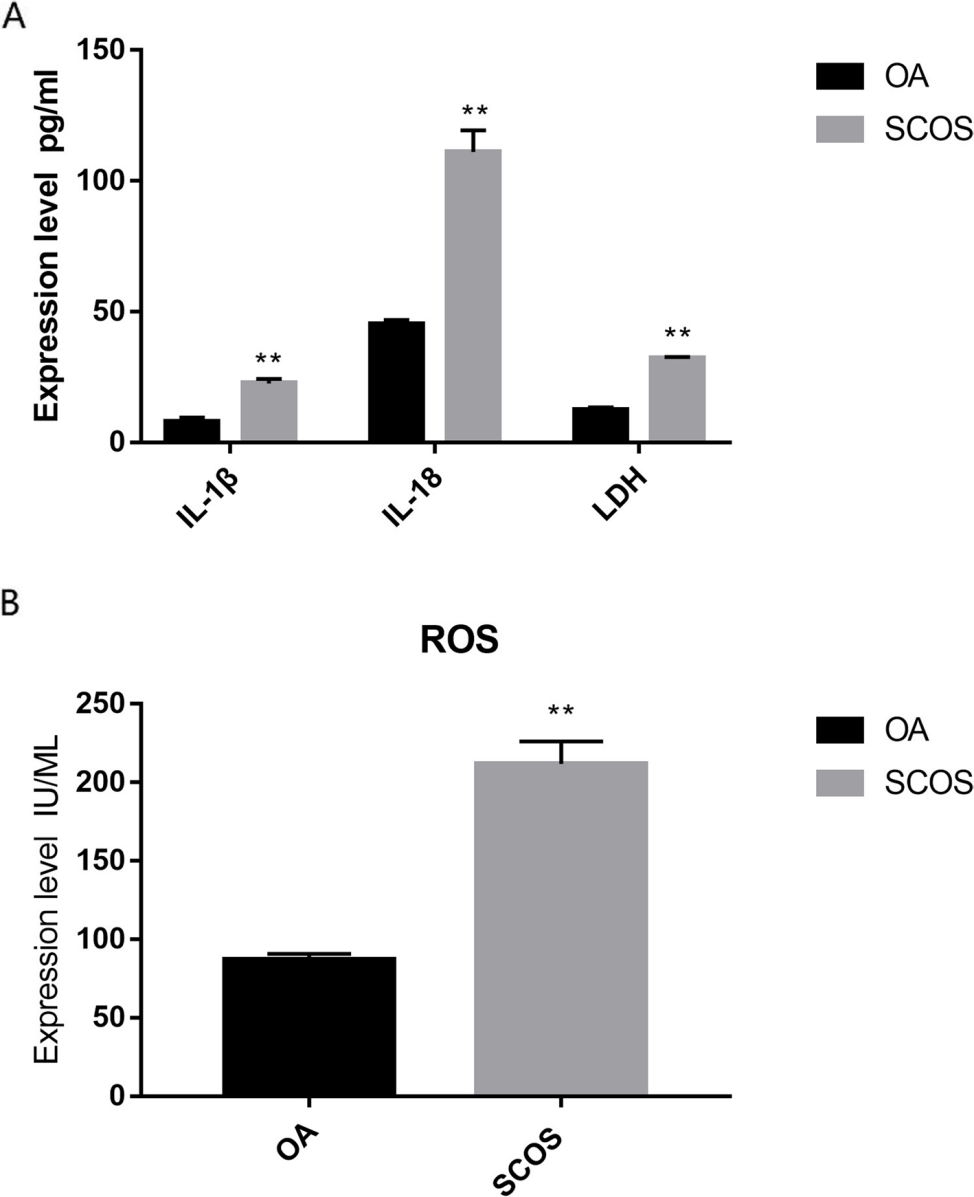
**A**,** B** Differential analysis of cell membrane-specific markers and inflammatory factors in cell pyroptosis. OA: Normal spermatogenesis group; SCOS: Sertoli cell-only syndrome group; Asterisks (**) indicate significant differences at *P* < 0.01 between the treated and control groups

CASP1 and CASP4 can activate GSDMD and GSDME to induce IL-1β and IL-18. At the same time, they can mediate cell membrane damage and release cellular contents and intracellular inflammatory cytokines. In our experiment, we found that LDH and ROS levels in the testicular tissue of patients with SCOS were significantly higher than those in normal testicular tissue. At the same time, we also found that IL-1β and IL-18 in the testicular tissues of patients with SCOS increased significantly, compared with normal testicular tissues, which further indicated that cell rupture and extensive inflammatory reactions occurred in SCOS(Fig. 8A, B).

## Discussion

SCOS is one of the most serious causes of male infertility [14]. Its pathological mechanisms and treatment are difficult in a clinical setting [15–18]. Apart from some known factors, such as Klinefelter syndrome, genomic AZF deletions, cryptorchidism, and mumps orchitis, which can lead to SCOS, there are many unknown causes [11]. The human testis transcriptome has been studied extensively using microarray assays in recent years, but no systematic studies haves reported mRNA profiling in human testicular tissue via high-throughput sequencing technology [19–21].

This study explored the gene expression profiles of human testicular tissues from patients with OA and SCOS using RNA-seq. We explored DEGs between these testicular tissues, aiming to discover key SCOS genes that underlie the molecular mechanisms of male infertility. In total, 6865 downregulated and 2541 upregulated genes were identified in the first round of analysis. In previous studies, *FANCM*, *TEX14*, *NR5A1*, and *WT1* were implicated as monogenic causes of SCOS, and *NANOS2*, *PLK4*, *WNK3*, and *FANCA* were reported in a single study and require additional cases to establish a monogenic causative link to SCOS [15]. Furthermore, our results showed significant differences in the expression of the above genes between patients with OA and SCOS (Table 2).

**Table 2:** Expression of previously identified SCOS-related genes in our study

Symbol	log2(FC)	*P*-value	FDR	Significance
*FANCM*	− 1.94022	0.000628	0.004049	down
*TEX14*	− 8.18966	3.22E-49	1.15E-46	down
*NR5A1*	2.146846	0.014571	0.052201	up
*WT1*	1.227602	0.032359	0.095337	up
*NANOS2*	− 5.52878	7.48E-05	0.000612	down
*PLK4*	− 4.22917	1.53E-18	6.02E-17	down
*FANCA*	− 3.92755	1.18E-15	3.54E-14	down

Considering the large number of DEGs screened in the first analysis, we conducted a second core DEG analysis based on the interaction network. Our study identified 21 downregulated and three upregulated key genes. RNA-seq data accuracy is the basis of all downstream pathway analyses [22–24]. Q-PCR analysis of all key genes showed consistent patterns with the RNA-seq data in this study.

GO enrichment analysis showed that key genes were mainly involved in the cell cycle, macromolecule metabolic process, cellular nitrogen compound metabolic process, and organelle organization for BP. Key genes primarily clustered in the nuclear, intracellular organelle, intracellular membrane-bounded organelle, and intracellular regions for CC. For MF, enzyme binding and nucleic acid binding were most enriched. Similarly, analysis of associated KEGG pathways showed that downregulated core genes were mainly involved in the cell cycle, progesterone-mediated oocyte maturation, and oocyte meiosis. Upregulated core genes were mainly related to cell pyroptosis and inflammation. There were 11 downregulated key genes in cell cycle pathways and nine downregulated key genes in progesterone-mediated oocyte maturation and oocyte meiosis pathways. Two more downregulated core genes were not concentrated in the same signalling pathway and were mainly involved in the activation of biological enzymes (Table 3).

**Table 3:** Functional analysis results of downregulated core differential genes

Function	Key genes
Cell cycle	*CDC6*, *CDC20*, *TUBA4A, TUBG1, CCNA1, CCNB1, CCNA2, CDT1, CDK1, CCNE1, MCM4*
Progesterone-mediated oocyte maturation and oocyte meiosis pathway	*BRCA1, E2F1, CHEK1, TOPBP1, MAD2L1, HIST3H3, BLM, UBE2C, MCM4*
Biological enzyme activation	*BIRC5*, *RAD51*

Downregulated genes were recently investigated, but no further research has been performed. It was generally believed that downregulated genes were likely caused by the loss of spermatogenic cells in SCOS. However, some scholars believe that downregulated genes could induce spermatogenesis dysfunction. This causal relationship is difficult to prove under experimental conditions. Therefore, this study mainly focused on upregulated key genes for further analysis.

We found that *CASP4*, *CASP1*, and *PLA2G4A* were upregulated in SCOS based on interaction networks. We compared upregulated core genes and found that both *CASP1* and *CASP4* are part of the caspase family, which participates in cell pyroptosis. Thus, we showed for the first time that cell pyroptosis might be involved in the SCOS pathological process.

In addition, immunohistochemical results showed that CASP1 and CASP4 in the normal spermatogenesis group were mainly expressed in the nuclei of spermatogenic, Sertoli, and interstitial cells. In the SCOS group, CASP1 and CASP4 were mainly expressed in the nuclei of Sertoli and interstitial cells owing to the loss of spermatogonia and spermatocytes in SCOS. At the same time, CASP1 and CASP4 expression levels in the testes of patients with SCOS were significantly higher than those in patients with normal spermatogenesis. We hypothesize that testis cell pyroptosis mediated by CASP1 and CASP4 could play an important role in modulating Sertoli cell functions and spermatogenesis.

Cell death occurs at any time in the process of individual growth and development. It is an ordinary life phenomenon in biology that plays an essential role in multicellular organism growth, development, and dynamic balance. There are two types of cell death: cell necrosis and programmed cell death, the latter including programmed cell necrosis and apoptosis. In addition, researchers have discovered new programmed cell death types in recent years, including cell swelling, autophagy, and pyroptosis. Unlike apoptosis and necrosis, pyroptosis depends on inflammatory caspases (CASP1 and CASP4/5/11), accompanied by an inflammatory response^[35]^. Therefore, pyroptosis plays an essential role in infectious, cardiovascular, and central nervous system diseases, as well as tumors [25–28]. Pyroptosis lies between apoptosis and necrosis. Pore formation in the cell membrane leads to cell integrity loss, cell content release, an increase in permeability, and inflammatory responses [29].

On one hand, moderate pyroptosis contributes to cell homeostasis and may effectively prevent excessive cell proliferation, which may protect the host. During pyroptosis, IL-1β and IL-18 secretion promotes leukocyte infiltration and activation. Cell lysis releases inflammatory mediators into the extracellular space, including ATP, IL-1β, and other heat-shock proteins that stimulate pro-inflammatory cytokine production by activating pattern-recognition receptors. This contributes to the control and ultimate resolution of microbial infection and allows tissues to return to their homeostatic state. On the other hand, high pyroptosis levels may induce inflammation, which is unfavorable to homeostasis maintenance in vivo. Excessive CASP1 and CASP4 activation can result in pyroptosis. Cells with excessive pyroptosis increase the levels of the inflammatory mediators IL-1β and IL-18, which can be detrimental to the host and may lead to disease if not controlled. Pyroptosis has been widely reported in various cell types, including macrophages, neutrophils, dendritic cells, endothelial cells, and cardiomyocytes. Mechanistically, pyroptosis is well preserved across cell types, involving the activation of canonical CASP1 and non-canonical CASP4/5/11 (human CASP4/5 and murine CASP11).

It is worth noting that canonical pyroptosis mediated by CASP1 and non-canonical pyroptosis mediated by CASP4 share the same downstream pathway regulated by GSDMD and GSDME. In our study, differential analysis of sequencing data also demonstrated high GSDMD and GSDME expression in SCOS. Furthermore, quantitative PCR results of testicular tissues also confirmed the increased expression of *GSDMD* and *GSDME* genes in patients with SCOS. To further explain the involvement of cell pyroptosis in SCOS occurrence and development, we also analyzed the protein expression level of pyroptosis-related genes. We found that the protein expression levels of CASP1, CASP4, GSDMD, and GSDME were significantly upregulated in the testicular tissues of patients with SCOS. Furtremore, CASP1 and CASP4 activity was significantly higher than that in patients with normal spermatogenesis. We also found that LDH, ROS, IL-1β, and IL-18 levels in the testicular tissue of patients with SCOS in our study were significantly increased. GSDMD and GSDME can induce inflammatory factors IL-1β and IL-18, cause cell membrane damage in testis cells due to pyroptosis. LDH and ROS can be rapidly released out of the cell when the membrane is damaged. Therefore, GSDMD and GSDME may be activated in patients with SCOS through CASP1 and CASP4 overexpression to promote testis cell pyroptosis.

We found that testicular cell pyroptosis might be a pathogenic basis of SCOS, but the specifics of the process remain unclear. It is well known that cell pyroptosis is closely related to inflammation and oxidative stress. Inflammation and oxidative stress are the most common activating factors and direct manifestations of cell pyroptosis [30]. We speculated that local inflammation and oxidative stress responses could promote testicular cell pyroptosis.

In addition, we identified a lot of inflammatory factors and ROS in the testicular tissues of patients with SCOS in this study. The inflammasome is upstream of the cell pyroptosis signaling pathway, which mainly contains receptor protein, adaptor ASC, and downstream CASP1\4\5\11. Activated CASP1\4\5\11 can promote the maturation and secretion of IL-1β and IL-18, leading to pyroptosis. Testicular inflammation can secrete various inflammatory factors, resulting in changes in spermatogenesis, sperm transport, and sperm function. IL-1β can activate intracellular inflammatory responses and further hinder the division and differentiation of spermatogonial stem cells. Previous studies have shown that ROS can promote pyroptosis-related genes and initiate cell pyroptosis. At the same time, oxidative stress produces ROS-activated inflammatory bodies, the most common upstream inflammatory response mechanism. Oxidative stress is an essential pathological factor in male infertility. The dynamic balance between ROS and antioxidants is related to male fertility. Thus, inflammation and oxidative stress could promote cell pyroptosis in SCOS.

In the case of external stimulation, moderate testis cell pyroptosis can clear the stimulus source. In contrast, excessive cell pyroptosis can lead to testis cell death and release inflammatory factors to increase the inflammatory response. Oxidative stress and inflammation in the testes of patients with SCOS could upregulate CASP1/CASP4 expression and promote pyroptosis. Overexpressed CASP1 could synthesize inflammatory mediators, such as IL-1β and IL-18, which could produce a testicular inflammatory response and disturb the differentiation and maturation of spermatogenic cells. Moreover, testis cell pyroptosis and its related inflammatory responses and oxidative stresses might interfere with the structure and function of blood-testis barrier and thus other harmful substances could enter the testis and break the spermatogenic process of the testis. For typical example, after infected by COVID-19 virus, the expression of interleukin IL-1β, IL-6 and tumor necrosis factor in testis were up-regulated, which induced inflammation, destroyed testicular tissue and interfered with spermatogenesis by interrupting the blood-testis barrier [31].

In this study, we ruled out some known factors, including chromosome disorders, Y chromosome microdeletions, cryptorchidism, radiation, cytotoxic drug intake, and mumps orchitis. Furthermore, we explored the pathogenic genes and pathogenesis of SCOS caused by other idiopathic factors based on RNA-seq. We did not exclude varicocele, testicular hydrocele, testicular microlithiasis, and other unknown factors when screening testicular tissues. Abnormal spermatogenesis in cryptorchidism is mainly caused by temperature-induced apoptosis and a large number of inflammatory factors. In mumps orchitis, local inflammation of the testes destroys spermatogenic cell structure and thus significantly reduces spermatogenic function. Varicocele mainly affects the secretory function of Sertoli cells through ROS and other injuries, which can change the spermatogenic microenvironment and induce spermatogenic cell loss. Testicular hydrocele and testicular microlithiasis mainly produce local inflammatory reactions and induce spermatogenesis dysfunction. The testis cell pyroptosis mediated by CASP1 and CASP4 found in this study was limited to the testicular tissue of patients with SCOS, possibly caused by known factors, such as varicocele, testicular microlithiasis, and testicular hydrocele or other unknown factors. Accumulated inflammatory factors, oxidative stress, and possible testis cell pyroptosis were also observed in the testicular tissue of patients with SCOS, caused by known factors, such as cryptorchidism and mumps orchitis.

In conclusion, for the first time, we found that the activities and expressions of CASP1 and CASP4, their key downstream proteins GSDMD and GSDME, and the inflammatory factors IL-1β, IL-18, LDH, and ROS were significantly increased in the testes of patients with SCOS. In turn, this could promote testis cell pyroptosis and further reduce spermatogonia, spermatocytes, and even spermatogonial stem cells during spermatogenesis in SCOS. Hence, we speculated that testis cell pyroptosis mediated by CASP1 and CASP4 might be a cause of SCOS pathogenesis.

## Supplementary Information

**Additional file 1.** 

## Data Availability

The datasets supporting the conclusions of this article are available from the authors on reasonable request. Please contact liuwantao2017@163.com.
