# Supplementary figures and images for: Testis cell pyroptosis mediated by CASP1 and CASP4: possible sertoli cell-only syndrome pathogenesis
Source: Reprod Biol Endocrinol. 2023 Jun 9;21:53. doi: 10.1186/s12958-023-01101-w (PMC10250861; doi:10.1186/s12958-023-01101-w)

**Fig. 7. Original image**


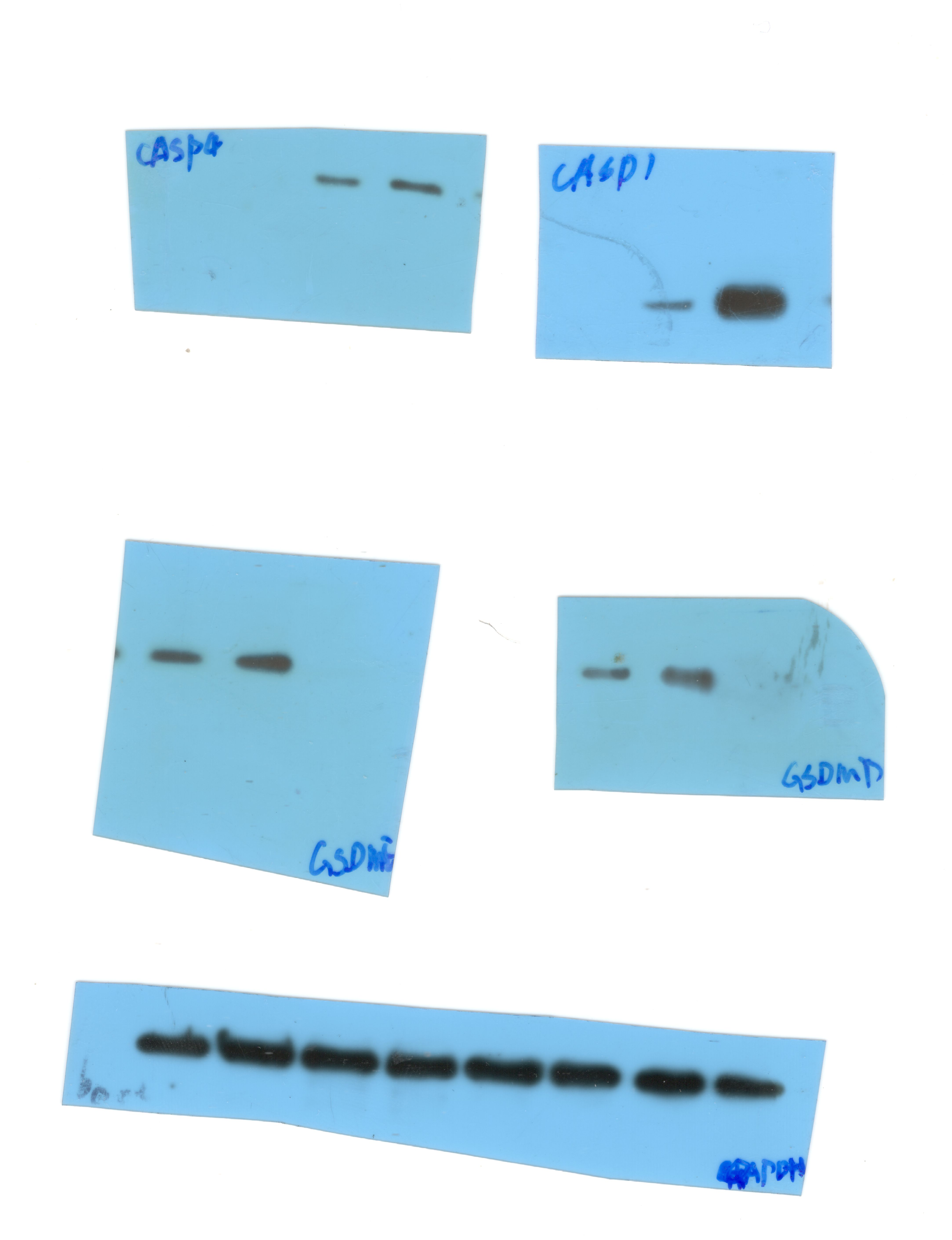

Supplement: Supplementary file 1 — Additional file 1. [file 12958_2023_1101_MOESM1_ESM.doc]
